# Investigating discrepancies in accuracy, agreement and interpretability for single-frame embryo classification tasks conducted by embryologists and deep learning models

**DOI:** 10.3389/frph.2026.1778326

**Published:** 2026-03-03

**Authors:** Radhika Kakulavarapu, Erwan Delbarre, Akriti Sharma, David Jahanlu, Michael A. Riegler, Trine B. Haugen, Mario Iliceto, Mette H. Stensen

**Affiliations:** 1 Department of Life Sciences and Health, Faculty of Health Sciences, OsloMet – Oslo Metropolitan University, Oslo, Norway; 2 Volvat Spiren, Oslo, Norway; 3 Simula Research Laboratory, Oslo, Norway; 4 Department of Social Work, Faculty of Social Science, Child Welfare and Social Policy, OsloMet – Oslo Metropolitan University, Oslo, Norway

**Keywords:** accuracy, agreement, deep learning, embryo assessment, interpretability

## Abstract

**Introduction:**

Artificial intelligence tools show promise in supporting clinical decision making, but their safe use requires evaluation of not only accuracy, but also agreement with experts and interpretability of model decisions. The aim of this study was to evaluate the accuracy and agreement of human embryologists and deep learning models in embryo stage classification, and to explore interpretability through explainable artificial intelligence.

**Methods:**

A retrospective, single-center study used single-frame embryo images (*n* = 245) classified according to developmental stage by three embryologists and two deep learning models, ResNet-34 and VGG16. Accuracy and agreement among all operators was evaluated, along with an assessment of interpretability with regards to model-generated explanations for spatial attention.

**Results:**

Embryologists achieved higher accuracy (89.9%) than ResNet-34 (78.8%, *p* < 0.001) and VGG16 (74.3%, *p* < 0.001), while overall agreement with the reference standard remained excellent for all operators (*κ*≥0.932). Stage-wise agreement was consistently stronger among embryologists than DL models (*κ* = 0.778–0.952 vs. 0.385–0.681). ResNet-34 Grad-CAMs were rated biologically relevant more often than VGG16 (89% vs. 59%, *p* < 0.001), yet interpretability did not consistently align with accuracy. Analysis of spatial overlap between model generated explanations was weak and observed to be lowest at the blastocyst stage, despite perfect model accuracy.

**Conclusions:**

These findings highlight the need for evaluation frameworks that integrate accuracy, agreement and interpretability to support safe and transparent development of artificial intelligence tools in assisted reproduction technology.

## 1 Introduction

Consistent monitoring of embryo development, whether conducted at discrete time-points or with the aid of time-lapse technology (TLT), represents a routine and critical task within assisted reproductive technology (ART). Considering the established relationship between embryo morphology and reproductive success (1–3), a visual assessment of embryo morphology is paramount towards the birth of a healthy child. To this end, TLT systems have enhanced the descriptive resolution of embryo monitoring, leading to its widespread clinical adoption (4–6). However, despite these advancements, morphological assessments remain time-consuming (7) and prone to variability across clinics (8), protocols (9, 10) and operators (11). As a means of addressing these limitations, several commercially available artificial intelligence (AI) systems have experienced a sharp increase in utilization (12–14), with demonstrated improvements in cost- and time-efficiency within clinical settings (15). Indeed, the application of various deep learning (DL) models, particularly convolutional neural networks (CNN) have been successfully implemented in classification tasks (16–20), embryo ranking strategies (21, 22), prediction of morphokinetics (23) and reproductive viability (24–26). However, as numerous studies suggest the equal (27–29), if not improved (25, 30) predictive performance of DL models compared to embryologists, reaching a clinical consensus on model evaluation remains an ongoing effort.

Model performance is often evaluated based on accuracy, which provides a measure of how closely a model prediction aligns with a known outcome (31). While reflective of some clinical tasks, such as embryo stage classification, several publications acknowledge the misleading properties of reporting model accuracy alone, particularly within the imbalanced datasets available in medical science (31–33). Consequently, as AI-powered tools gain traction within the field of ART, models are being evaluated with a greater range of performance indicators (5, 31, 34), including a lens of agreement (21, 29, 35–37). Providing a measure of consistency to clinical gold-standards, benchmarking DL model performance against embryologist performance has become a common method in rationalizing the use of AI systems within *in-vitro* fertilization (IVF) laboratories (13). Practically, however, a major barrier to adoption has been attributed to difficulties in interpreting how a model has reached a particular decision. Thus, the lack of transparency within “black box” DL architectures, where decisions can carry significant ethical, emotional and medical implications, leads to mistrust, misinterpretation and poor clinical integration (38, 39). Moreover, an incomplete understanding of model predictions raises concerns about whether predictions are based on biologically meaningful features, or spurious correlations (40). Remarkably, very few studies have examined whether embryologists and DL models reach the same decisions based on shared interpretive features (36), thereby representing a notable gap in the effective evaluation of clinical AI (41).

Building upon these observations, explainable artificial intelligence (XAI) is emerging to bridge the gap between accuracy and agreement (5, 38, 42–44), a distinction that is critical when model classifications must be justified or explained. XAI methods aim to increase interpretability by visualizing regions or features that contribute strongly to resultant predictions (43, 45). One such method, Gradient-weighted Class Activation Mapping (Grad-CAM), generates local, gradient-based saliency maps, known as heatmaps, that visualize model attention upon classification tasks (46–48). Another occlusion-based approach perturbs the input image to create Local Interpretable Model-agnostic Explanations (LIME), which outline salient input regions that contribute towards changes in the class of a given model (19, 49). Despite the growing interest in XAI, applications of Grad-CAM (37, 47, 48, 50–52), LIME (53, 54), or a combination of XAI methods (19, 49, 51) still lack comprehensive evaluation by domain experts. In fact, most existing work focuses on predictive outcomes such as blastocyst quality or implantation potential, without deeper evaluation of spatial attention patterns (13, 15). Consequently, reports that demonstrate alignment between model accuracy, agreement and domain expert interpretability remain scarce.

This study aims to address these gaps by evaluating the accuracy and agreement of clinical embryologists and two DL models, ResNet-34 and VGG16, in the classification of single-point images representing embryonic developmental stages. The choice of DL models in this study is based on our previous publication (49) and represents some of the most frequently used architectures for embryo development and assessment tasks (13). Crucially, beyond model performance alone, we apply Grad-CAM to each model, thereby creating “explanations” that are evaluated for biological relevance and interpretability by embryologists. Additionally, we apply LIME explanations to the same models in order to quantitatively evaluate the degree of spatial overlap between independent XAI techniques. The selection of local, *post-hoc* XAI methods such as Grad-CAM and LIME are intended to cater to the equally local or individual differences observed within embryo cohorts, thereby allowing insight into specific predictions, rather than global markers. By investigating classification patterns, domain expert assessments and explainability-based evaluations, this study aims to provide deeper insight into the reliability of AI-driven embryo stage classification, thereby contributing to the responsible development and implementation of AI tools in ART.

## 2 Methods

### 2.1 Data collection

Data from 531 embryos were obtained retrospectively from 526 women undergoing assisted reproductive treatment (ART) at a single fertility clinic in Oslo, Norway, between October 2013 and February 2019. All fertilized zygotes, inseminated by either *in-vitro* fertilization (IVF) or intracytoplasmic sperm injection (ICSI), were placed inside individual culture chambers within an EmbryoScope™ (Vitrolife, Denmark) time-lapse incubator (5% O_2_, 6% CO_2_, 89% N_2_). Embryos were cultured and monitored within the EmbryoScope™ for a maximum of 5 days. Annotations were manually performed by three separate operators using EmbryoViewer™ (Vitrolife, Denmark) and denoted the following stages: start of 2-cell (t2), 3-cell (t3), 4-cell (t4), 5-cell (t5), 8-cell (t8), 9-cell stage (t9), morula stage (tM), and formation of a full blastocyst (tB). Manual annotations by embryologists made use of all available focal planes during routine embryo assessment, consistent with current guidelines for good practice (55). These annotations were used to identify the corresponding frame number, thereby informing the extraction of frames that were morphologically appropriate and unambiguous to each developmental stage of interest. As described previously in Sharma et al. (49), in order to make the dataset more robust, frames from the central and peripheral focal planes were randomly extracted 1–3 frames after the annotated timing and used for model training. The independent test set contained images of embryos at the central plane only.

### 2.2 Deep learning algorithms

**Figure 1:**
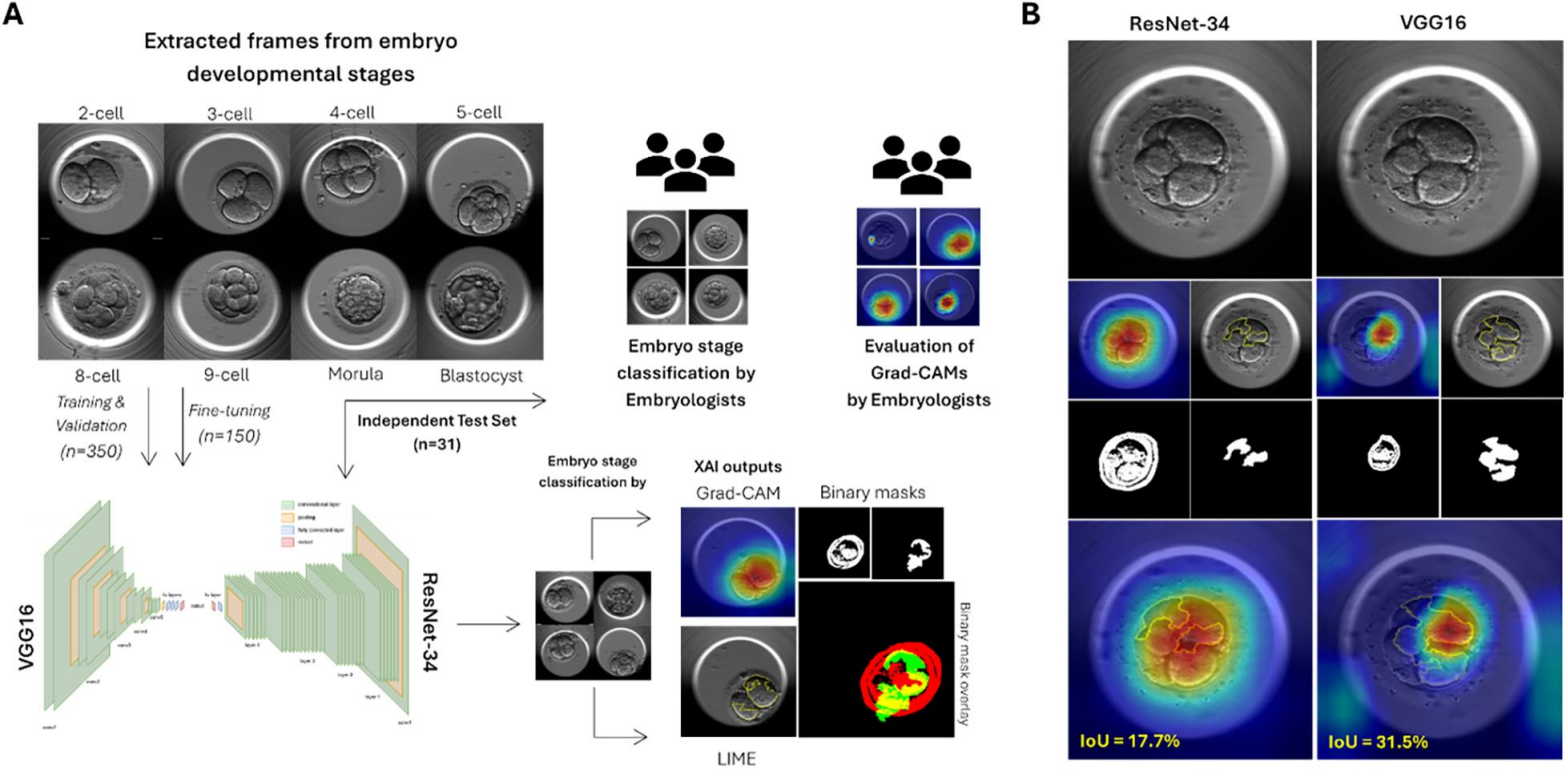
Overview of methodology. **(A)** Data distribution and methodology. Extracted frames representing stages of embryo development from 500 individual embryos, including the 2-cell, 3-cell, 4-cell, 5-cell, 8-cell, 9-cell, morula and blastocyst stages, were used to train, validate and fine-tune ResNet-34 and VGG16. An independent test set of extracted frames representing the remaining 31 embryos were presented to three embryologists, VGG16 and ResNet-34 for classification of embryo development stages. Further generation of explainable outputs by each DL model was conducted by means of Grad-CAM and LIME. Grad-CAM outputs were additionally evaluated by the same embryologists for relevance to biologically relevant areas. **(B)** Visualization of overlap between XAI outputs using binary masks. Each frame presented to ResNet-34 and VGG16 generated explainable outputs using Grad-CAM and LIME. Binary masks were created using a custom python script from each XAI output, per frame, and were overlayed to calculate intersection over union (IoU). IoU is presented as a proportion (%) for each frame.

ResNet-34 and VGG16 were used in this study based on calculated performance metrics (Supplementary Table S1), the training and fine-tuning of which is previously described (49). Briefly, 8-bit images (500 × 500) were obtained from the EmbryoScope™ and resized for both models (224 × 224). ImageNet base weights were utilized, and fine-tuning was conducted using the Adam Optimizer (learning rate 0.001). Standard normalization was applied, and no data augmentation techniques were used. For model training, images were extracted from a total of 350 embryos, from which 335 successfully developed to the blastocyst stage. Likewise, images were extracted from 150 different embryos for fine-tuning, and 31 embryos for the independent test set (Figure 1A). Of note, embryos were distributed across each dataset prior to extraction of developmental stage frames to ensure the same embryo did not recur, thereby preventing data leakage. All classification results from this study represent data from the independent test set alone.

### 2.3 Embryologists' evaluation of Grad-CAM outputs

To assess the spatial focus of DL models in classification tasks, features used to classify each image were visualized using Grad-CAM, the configuration of which is previously described (49). The resultant color grading ranged from areas of high relevance (red) to low relevance (blue), for each model. These Grad-CAM outputs were independently evaluated by three embryologists (Figure 1A), each of whom rated the relevance of each Grad-CAM computation as “good” (identified areas of high relevance are localized within biologically relevant structures/areas), “poor” (identified areas of high relevance did not correspond to the correct or any biologically relevant structures/areas), or “intermediate” (identified areas of high relevance corresponded to both relevant and irrelevant biological structures/areas). These assessment groups were developed following several focused discussions with embryologists that considered the range of patterns displayed by each Grad-CAM output and were re-grouped into simplistic assessment criteria following operator consensus (Supplementary Material 2). Embryologists were blinded to the classification result by the DL models. Proportions of “good”, “intermediate” and “poor” assessments were calculated per developmental stage for each DL model.

### 2.4 LIME explanations and calculation of spatial overlap

All images from the independent test set were further assessed using Local Interpretable Model-agnostic Explanations (LIME), the configuration of which is previously described (49). Only regions contributing positively to the predicted embryo stage were generated, where super pixels were denoted by yellow boundaries. To ascertain the extent of spatial overlap between both XAI techniques, binary masks were created for each explanation (Figure 1B). A custom Python script was created to process Grad-CAM and LIME outputs, generate binary masks and calculate intersection over union (IoU), available on GitHub (Faiga91/IoU-Interpretability-Analysis; commit 065de77). For Grad-CAM masks, color segmentation was used to establish higher and lower bounds corresponding to red and cyan hues, respectively. In this manner, the range of pixels within the identified color bounds were masked as white, corresponding to contributive values. On the other hand, blue hues were considered non-contributive and remained black. For LIME masks, yellow super pixel contours were used to create a base mask, which was then filled and merged into a single, white mask. Pixels outside of the yellow boundaries were converted into black, non-contributive pixels. Thus, to ascertain the level of spatial overlap between each binary mask, an IoU score was calculated between 0 and 1. Values of 0 denoted no spatial overlap between Grad-CAM and LIME explanations, whereas values of 1 represented complete spatial overlap between XAI outputs. IoU values are presented as proportion (%) of overlap between XAI outputs.

### 2.5 Statistical analysis

All statistical analyses were conducted using IBM SPSS Statistics (version 29). Embryo-level predictions from each rater and model were encoded as ordinal variables ranging from 1 to 7, corresponding to the developmental stages: 2-cell, 3-cell, 4-cell, 5-cell, 8 or 9 cell, morula, and blastocyst, respectively. Upon initial classification assessment by embryologists, misclassification of 8-cell embryos as 9-cell embryos, and vice versa, was frequently observed. This pilot finding led to the merging of these groups to mimic clinical practice in the grading of these stages. Classification accuracy was calculated as the proportion of embryos correctly identified relative to the reference standard. Agreement between each rater/model and the reference standard was evaluated using quadratic-weighted Cohen's kappa (*κ*) and 95% confidence intervals for *κ* were estimated. Inter-rater reliability was assessed through pairwise quadratic-weighted Cohen's *κ* across all raters and models. Additionally, Fleiss' *κ* was calculated to summarize agreement among the three embryologists. Stage-specific performance was examined by computing accuracy within each developmental stage subgroup. Confusion matrices were generated to visualize misclassification patterns and identify stages with elevated error rates. Comparative analysis of overall classification accuracy among raters and models was performed using Cochran's *Q* test. Where significant differences were observed, *post-hoc* pairwise comparisons were conducted using McNamar's test, with Holm-adjusted *p*-values applied to control for multiple testing. Chi squared analyses were used to compare proportions of qualitative assessments. A Wilcoxon matched pairs signed-rank test was used to assess stage-wise differences in IoU, between models. *P*-values less than 0.05 were considered significant.

### 2.6 Ethical considerations

All data used in this study was anonymized and approved by the Regional Committee for Medical and Health Research Ethics – South-East Norway (2018/477, REC South-East).

## 3 Results

### 3.1 Accuracy observed across raters

**Figure 2:**
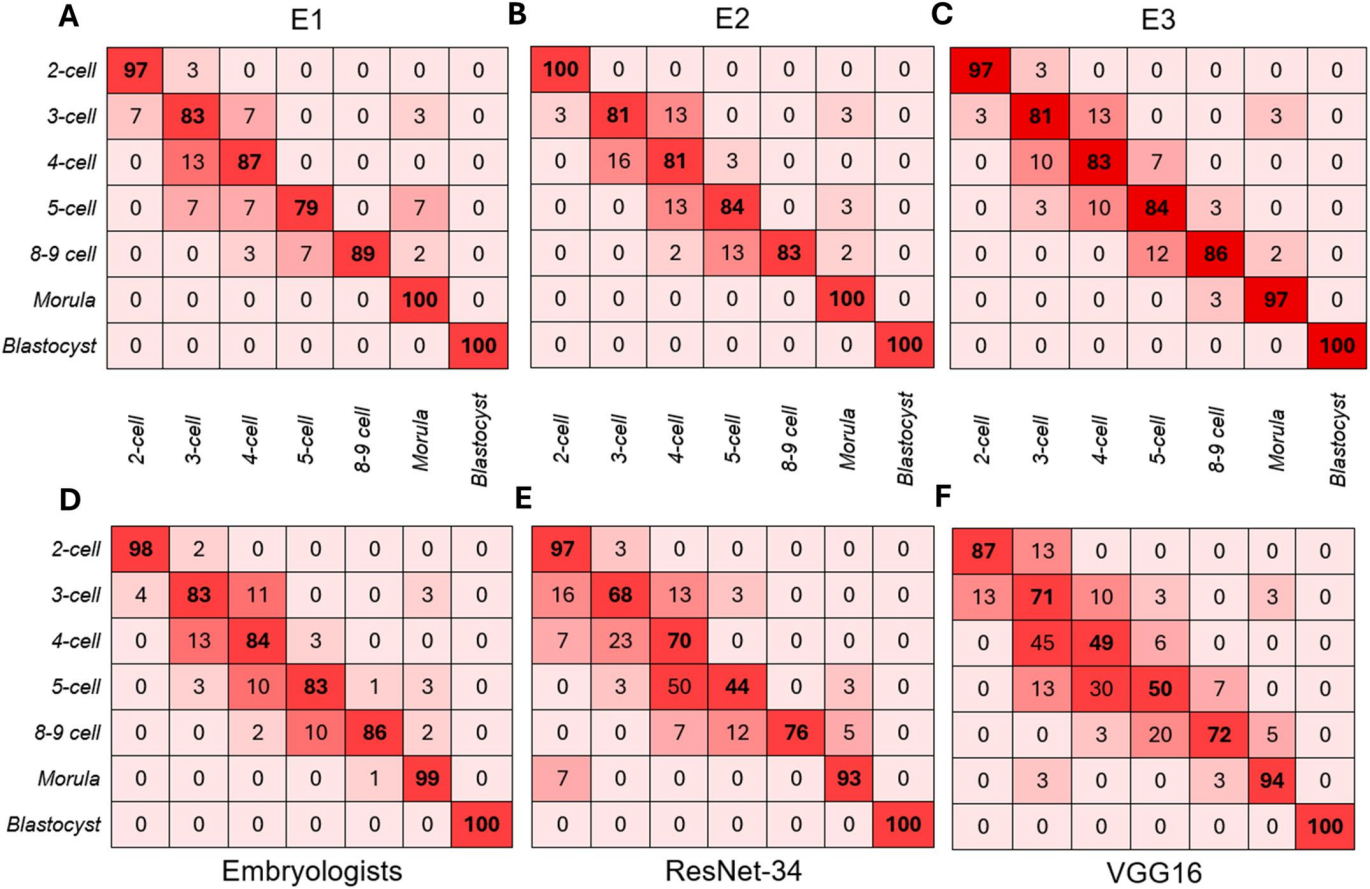
Confusion matrices for all raters. Each matrix shows the proportion of embryos in each true developmental stage that were classified into each predicted stage by **(A)** Embryologist 1 (E1), **(B)** Embryologist 2 (E2), **(C)** Embryologist 3 (E3), **(D)** All embryologists (E1, E2, E3 combined), **(E)** ResNet-34 and **(F)** VGG16. Darker shades of red indicate higher proportions of accuracy (correctly classified). Correct classifications are represented along the diagonal. while off-diagonal cells indicate misclassifications. Proportions are rounded up to the nearest whole value.

A total of 245 images representing various stages of embryo development from 31 embryos were assessed by three clinical embryologists (E1, E2, E3) and two DL models, ResNet-34 and VGG16. Collectively, embryologists accurately identified 89.9% (*n* = 661) of presented images according to developmental stage. In comparison, ResNet-34 and VGG16 accurately classified 78.8% (*n* = 193) and 74.3% (*n* = 182), respectively, evidently displaying higher error rates than human operators. Proportions of incorrect classifications were indeed found to be stage-dependent (Figure 2). Although all raters achieved perfect accuracy at the blastocyst stage and minimal errors at the morula stage, the highest misclassification rate within embryologists occurred at the 3-cell (18%) and 5-cell stages (17%). Notably, the majority of these misclassifications occurred within adjacent developmental stages, where 11% of 3-cell embryos and 10% of 5-cell embryos were misclassified as 4-cell embryos (Figure 2D). Similar trends in misclassification were observed for DL models, where 50% and 44% of 5-cell embryos were accurately identified with VGG16 and ResNet-34, respectively. Strikingly, adjacent cell-stage errors were observed at higher proportions for both DL models compared to embryologists, ranging from 3% to 50% for ResNet-34 (Figure 2E), and between 3% and 45% for VGG16 (Figure 2F). An overall significant difference in classification accuracy among the five operators was found [*Q*(4) = 60.77, *p* < 0.001]. Although *post-hoc* pairwise comparisons (Holm-adjusted) revealed no differences in accuracy between the three embryologists (E1–E2: *p* = 0.629; E1–E3: *p* = 0.845; E2–E3: *p* = 1.000), the accuracy of each embryologist differed from both deep learning models (*p* < 0.001). However, no differences in accuracy were found between ResNet-34 and VGG16 (*p* = 0.144).

### 3.2 Agreement between raters

Agreement with the reference standard was close to perfect for all raters and models (Table 1). Embryologists demonstrated slightly higher agreement with the reference standard (*κ* range: 0.969–0.976), however both DL models also showed excellent agreement (ResNet-34: *κ* = 0.932; VGG16: *κ* = 0.940). All *κ* values were statistically significant (*p* < 0.001), with overlapping confidence intervals between embryologists and models. Perfect agreement was observed at the blastocyst stage (*κ* = 1.000) for all raters, leading to its removal from the present analysis. For all remaining stages, agreement among the three embryologists was high, with an overall Fleiss' *κ* of 0.878 (95% CI: 0.848–0.908. *p* < 0.001), indicating excellent reliability across human operators. Fleiss' *κ* was only used to assess agreement among the three embryologists, whereas Cohen's *κ* was sufficient to make comparisons between two operators. As such, pairwise quadratic weighted Cohen's kappa between all operators indicated high agreement (Table 2).

**Table 1:** Quadratic-weighted Cohen's *κ* values between each rater/model and the reference standard (ground truth).

Operator	Weighted kappa (*κ*)	95%CI	*p*-value
E1	0.969	0.948–0.990	<0.001
E2	0.974	0.954–0.993	<0.001
E3	0.976	0.957–0.995	<0.001
ResNet-34	0.932	0.893–0.971	<0.001
VGG16	0.940	0.912–0.968	<0.001

Embryologists (E1, E2, E3) and deep learning models (ResNet-34, VGG16) displayed excellent agreement to ground truth.

**Table 2:** Pairwise quadratic-weighted Cohen's κ values between and within operators.

Comparison of operators	Weighted kappa (κ)	95%CI	*p*-value
E1-E2	0.974	0.958–0.991	<0.001
E1-E3	0.974	0.960–0.988	<0.001
E2-E3	0.983	0.975–0.991	<0.001
ResNet-E1	0.915	0.871–0.959	<0.001
ResNet-E2	0.928	0.885–0.970	<0.001
ResNet-E3	0.926	0.884–0.968	<0.001
VGG-E1	0.930	0.899–0.960	<0.001
VGG-E2	0.938	0.910–0.967	<0.001
VGG-E3	0.934	0.901–0.966	<0.001
VGG-ResNet	0.937	0.899–0.976	<0.001

Embryologists (E1, E2, E3) and deep learning models (ResNet, VGG). Kappa (κ) values are presented for each comparison, along with 95% confidence intervals (CI) and the corresponding *p*-value.

**Figure 3:**
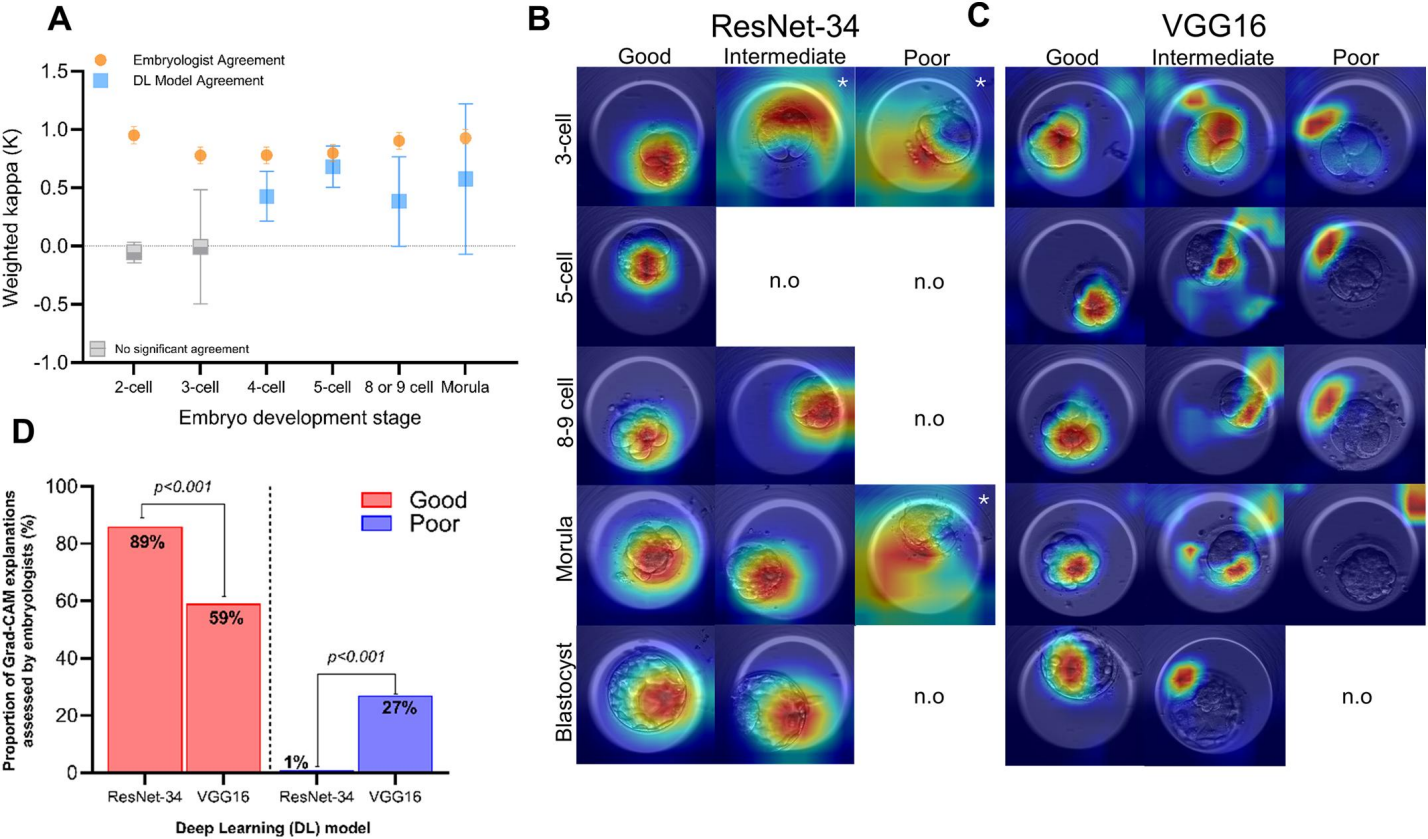
Agreement and interpretability analyses. **(A)** Stage-wise agreement of embryo classification. Fleiss’ multirater kappa (*κ*) was used to assess stage-wise classification agreement among the three embryologists. Cohen's weighted kappa (*κ*) was used to assess stage-wise classification agreement between ResNet-34 and VGG16. Kappa (*κ*) values are denoted by orange circles for embryologists, and blue squares for deep learning (DL) models. Error bars represent 95% confidence intervals for each *κ* value. Grey squares represent analyses where the calculated *κ* value was not found to be significant (*p* > 0.05). **(B)** Grad-CAM explanations generated by ResNet-34. Examples of Grad-CAM outputs generated by ResNet-34 are presented in each row for the 3-cell, 5-cell, 8- or 9-cell embryos, morulae and blastocysts. Columns display one example of embryos evaluated as “good”, “intermediate” or “poor” for each relevant embryo stage. If an “intermediate” or “poor” evaluation was not assigned at any particular stage, the figure states “not observed” (n.o). White asterisks on selected frames indicate only one observation of the Grad-CAM evaluation at the relevant embryo development stage. **(C)** Grad-CAM explanations generated by VGG16. Examples of Grad-CAM outputs generated by VGG16 are presented in each row for the 3-cell, 5-cell, 8- or 9-cell embryos, morulae and blastocysts. Columns display one example of embryos evaluated as “good”, “intermediate” or “poor” for each relevant embryo stage. If an “intermediate” or “poor” evaluation was not assigned at any particular stage, the figure states “not observed” (n.o). **(D)** Overall evaluation of Grad-CAM explanations by embryologists. The overall proportion of Grad-CAM explanations evaluated as “good” and “poor” for each deep learning (DL) model. Red bars represent “good” evaluations from ResNet-34 and VGG16, whereas blue bars represent “poor” evaluations by both models. Proportions (%) are listed at the top of each bar. *P*-values were calculated using a chi-square analysis and are displayed using comparison lines.

Stage-specific agreement among embryologists was consistently strong, ranging from 0.778 (95% CI: 0.706–0.851, *p* < 0.001) for 3-cell embryos to 0.952 for 2-cell embryos (95% CI: 0.879–1.024, *p* < 0.001) (Figure 3A). In contrast, negative and non-significant *κ* values were found upon 2-cell (*κ* = −0.054, 95% CI: −0.143–0.034, *p* = 0.696) and 3-cell embryo classification by DL models (*κ* = −0.005, 95% CI: −0.496–0.485, *p* = 0.975), suggesting insufficient evidence of agreement between ResNet-34 and VGG16 within the earliest embryo divisions. On the other hand, moderate agreement between the DL models was observed for 4-cell embryos (*κ* = 0.427, 95% CI: 0.214–0.640, *p* = 0.015) onwards. Notably, DL models displayed substantial agreement at best, upon classifying the 5-cell stage embryo (*κ* = 0.681, 95% CI: 0.503–0.860, *p* < 0.001). Nonetheless, Cohen's weighted *κ* values were found to be consistently lower than *κ* values for embryologist agreement at all stages of embryo development, and with larger confidence intervals (Supplementary Table S3).

### 3.3 Interpretability of Grad-CAM explanations

Grad-CAM outputs were generated by ResNet-34 and VGG16 (Figure 3B). These outputs were presented to embryologists and assessed for relevance to biologically appropriate areas as “good”, “intermediate” or “poor”. Overall, we observed greater proportions of “good” classifications assigned to ResNet-34 Grad-CAM outputs (89%) compared to those generated VGG16 (59%, *p* < 0.001, Figure 3D). Notably, very few Grad-CAM explanations (1%, *n* = 3) stemming from ResNet-34 were evaluated as “poor”. In fact, a larger proportion of VGG16 Grad-CAM outputs were assessed as “poor” (27%, *p* < 0.001), suggesting that embryologists interpreted ResNet-34 to focus on more biologically relevant areas within embryo images, compared to VGG16.

#### 3.3.1 Stage-wise assessment of Grad-CAM outputs

**Figure 4:**
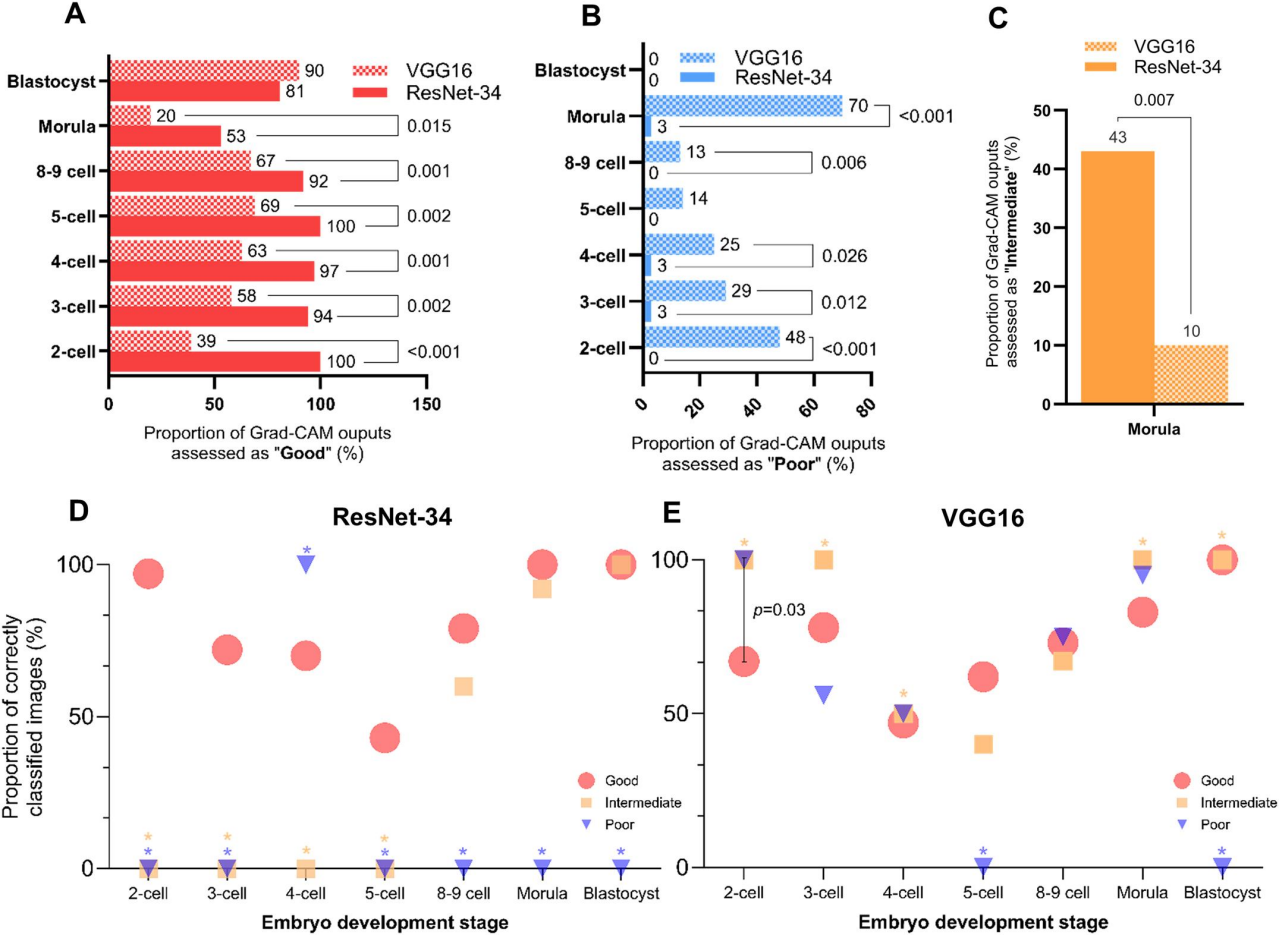
Evaluation of biological relevance and resultant accuracy. **(A)** “Good” Grad-CAM evaluations according to embryo stage. Bars indicate proportion (%) of images in each stage of embryo development that were assessed by embryologists for relevance of biological focus. Solid red bars represent Grad-CAM explanations generated by ResNet-34, while checkered bars represent VGG16 Grad-CAM explanations. Proportions are listed to the right of each relevant bar. *P*-values are listed using comparison lines for significant comparisons only, assessed using chi-squared tests. No comparison lines or *p*-values are listed where *p* > 0.05. **(B)** “Poor” Grad-CAM evaluations according to embryo stage. Bars indicate proportion (%) of images in each stage of embryo development that were assessed by embryologists for relevance of biological focus. Solid blue bars represent Grad-CAM explanations generated by ResNet-34, while checkered bars represent VGG16 Grad-CAM explanations. Proportions are listed to the right of each relevant bar. *P*-values are listed using comparison lines for significant comparisons only, assessed using chi-squared tests. No comparison lines or *p*-values are listed where *p* > 0.05. **(C)** “Intermediate” Grad-CAM evaluations at the morula stage. Bars indicate proportion (%) of images in each stage of embryo development that were assessed by embryologists for relevance of biological focus. Solid orange bars represent Grad-CAM explanations generated by ResNet-34, while checkered bars represent VGG16 Grad-CAM explanations. Proportions are listed above each relevant bar. *P*-values are listed using comparison lines, assessed using chi-squared tests. **(D)** Proportions of correctly classified images by ResNet-34 according to spatial focus group. Red circles represent the proportion of images that were classified as “good” by embryologists and correctly classified by ResNet-34. Orange squares represent the proportion of images that were classified as “intermediate” by embryologists and correctly classified by ResNet-34. Blue triangles represent the proportion of images that were classified as “poor” by embryologists and correctly classified by ResNet-34. Asterisks colored in orange or blue indicate too few instances to allow statistical comparison for the proportion of embryo images evaluated as “intermediate” or “poor”, respectively. No comparison lines or *p*-values are listed where *p* > 0.05. **(E)** Proportions of correctly classified images by VGG16 according to spatial focus group. Red circles represent the proportion of images that were classified as “good” by embryologists and correctly classified by VGG16. Orange squares represent the proportion of images that were classified as “intermediate” by embryologists and correctly classified by VGG16. Blue triangles represent the proportion of images that were classified as “poor” by embryologists and correctly classified by VGG16. Asterisks colored in orange or blue indicate too few instances to allow statistical comparison for the proportion of embryo images evaluated as “intermediate” or “poor”, respectively. *P*-values are listed using comparison lines for significant comparisons only. No comparison lines or *p*-values are listed where *p* > 0.05.

Across all stages of development, with the exception of the blastocyst stage, embryologists assessed ResNet-34 Grad-CAMs as “good” at higher proportions than VGG16 generated Grad-CAM explanations (Figure 4A; Supplementary Table S4). The proportion of blastocyst-stage embryos assessed as “good” were comparable between ResNet-34 (91%) and VGG16 (90%). In contrast, “poor” assessments were observed at higher proportions for VGG16 generated Grad-CAM outputs than ResNet-34 Grad-CAM outputs for 2-cell (48% vs. 0%, *p* < 0.001), 3-cell (29% vs. 3%, *p* = 0.012), 4-cell (25% vs. 3%, *p* = 0.026) and 8- or 9-cell embryos (13% vs. 0%, *p* = 0.006) (Figure 4B). Strikingly, 70% of VGG16 generated Grad-CAM outputs were assigned as “poor” for morula stage embryos in particular, indicating a majority of all VGG16 Grad-CAM outputs did not focus on biologically meaningful areas upon morula stage classification. Likewise, proportions of “intermediate” assessments did not vary between ResNet-34 and VGG16 for any embryo stages, except the morula stage (Figure 4C; Supplementary Table S4). In fact, a higher proportion of ResNet-34 generated Grad-CAM explanations for the morula were assigned as “intermediate” (43%), compared to VGG16 (10%, *p* = 0.007). On the other hand, Grad-CAM explanations at the blastocyst stage presented from either model did not result in any “poor” evaluations from embryologists.

To further ascertain whether domain expert evaluation of spatial focus aligned with observed accuracy, the proportion of correctly classified images assessed as “good”, “intermediate” and “poor” were illustrated for ResNet-34 and VGG16 (Figures 4D,E). Indeed, our findings illustrate greater accuracy within “good” ResNet-34 Grad-CAM outputs, particularly for 2-cell (97%) and 3-cell (72%) embryos (Figure 4D). However, despite 100% of 5-cell stage ResNet-34 Grad-CAMs being evaluated as “good” by embryologists, only 43% of these instances were accurately classified. Moreover, there were no observed differences in accuracy between ResNet-34 Grad-CAMs assessed as “good” or “intermediate” for the 8- or 9-cell stage (79% vs. 60%, *p* = 0.322), morula stage (100% vs. 92%, *p* = 0.448) or blastocyst stage (100% vs. 100%, *p* > 0.999).

Remarkably, greater accuracy was observed for “poor” VGG16 generated Grad-CAM outputs at the 2-cell stage (100%) compared to “good” outputs (67%, *p* = 0.031, Figure 4E). Although this was the only comparison indicating a statistically significant difference in accuracy between spatial focus groups, comparable proportions of accuracy were consistently observed at the 3-cell (Good: 78% vs. Poor: 56%, *p* = 0.372), 4-cell (Good: 47% vs. Poor: 50%, *p* > 0.999) and 5-cell stages (Good: 62% vs. Intermediate: 40%, *p* = 0.613). Notably, 95% of morula-stage Grad-CAMs evaluated with “poor” biological relevance were correctly classified, compared to 83% accuracy within frames assessed as “good”. Similarly, proportions of accurately classified frames within “good” (73%), “intermediate” (67%) and “poor” (75%) assessments were comparable. Moreover, 100% accuracy was observed at the blastocyst stage by both ResNet-34 (Figure 4D) and VGG16 (Figure 4E) regardless of qualitative assessment as “good” or “intermediate”.

### 3.4 Spatial overlap with LIME explanations

**Figure 5:**
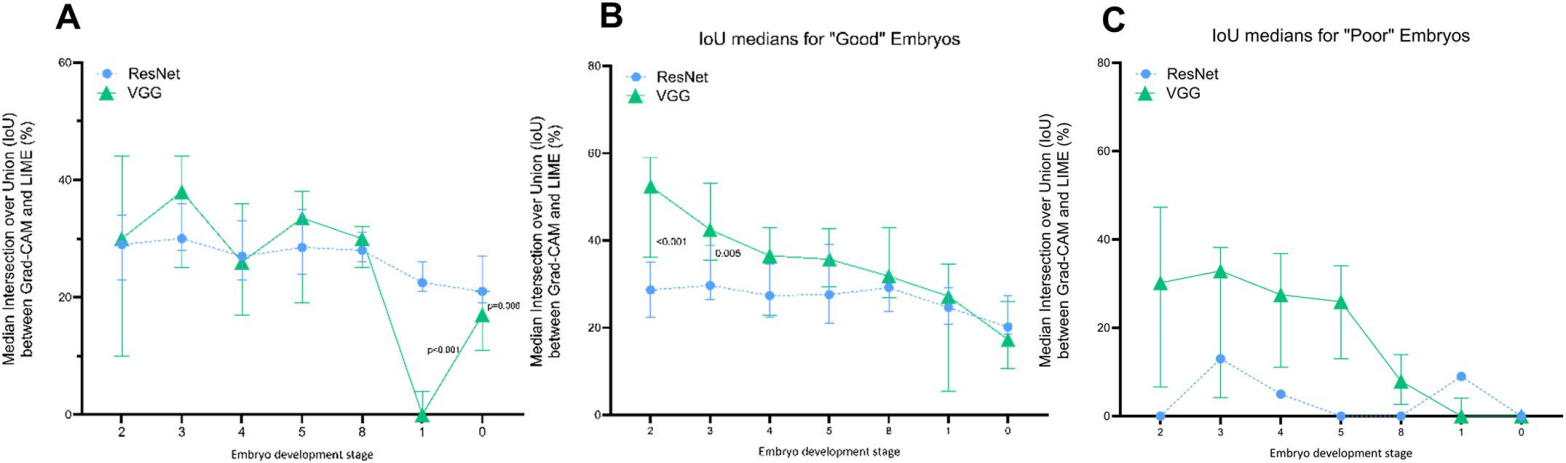
Intersection over union (IoU) between grad-CAM and LIME explanations. Median IoU distribution among embryo development stages. Embryo development stages listed along the *x*-axis are ordinally coded to represent the 2-cell embryo (2), 3-cell embryo (3), 4-cell embryo (4), 5-cell embryo (5), 8- or 9-cell embryo (8), morula (1) and blastocyst (0). Blue circles represent median IoU values calculated for ResNet-34 generated explanations, whereas green triangles represent median IoU values calculated for VGG16 generated explanations. Error bars for each node represent 95% confidence intervals. *P*-values were calculated using a Wilcoxon matched pairs signed-rank test and listed using comparison lines between significantly different nodes only. No comparison lines or *p*-values are listed where *p* > 0.05. **(A)** Overall median IoU distribution per embryo stage. **(B)** Median IoU distribution for Grad-CAM outputs evaluated as “good”. **(C)** Overall median IoU distribution per embryo stage. **(B)** Median IoU distribution for Grad-CAM outputs evaluated as “good”. Statistical tests could not be reliably conducted as too few ResNet-34 Grad-CAM outputs were assessed as “poor”.

Binary masks were generated to calculate intersection over union (IoU) scores and quantitatively assess the degree of overlap between Grad-CAM and LIME explanations. A Wilcoxon matched-pairs signed-rank test demonstrated higher IoU for ResNet-34 explanations (26.6%, 95% CI: 25.4–28.4) compared to VGG16 (25.2%, 95% CI: 21.0–28.8, W = −6,091, median difference = −0.0236, *p* = 0.006), suggesting Grad-CAM and LIME outputs from ResNet-34 were more spatially consistent across XAI methods, compared to VGG16. Yet, upon investigating the median distribution of IoU between embryo development stages (Figure 5A), we found no significant differences in median IoU between models among any of the cleavage stages (2-cell to 8- or 9-cell embryos). On the other hand, spatial overlap between VGG16 generated XAI outputs was significantly lower at both the morula (0%, 95% CI: 0.0–4.0, *p* < 0.001) and blastocyst stages (17.0%, 95% CI: 11.0–21.0, *p* = 0.006) compared to ResNet-34 generated outputs (*Morula*: 22.5%, 95% CI: 21.0–26.0; *Blastocyst*: 21.0%, 95% CI:19.0–27.0).

Furthermore, Grad-CAM explanations evaluated as “good” were assessed for their corresponding overlap with LIME explanations, per developmental stage (Figure 5B). Despite higher proportions of ResNet-34 Grad-CAMs being evaluated as “good” by embryologists, median IoU scores indicated less spatial overlap with corresponding LIME explanations, compared to VGG16 counterparts. Grad-CAM outputs generated by VGG16 at the 2-cell stage indicated the highest spatial overlap with LIME (52.4%, 95% CI: 36.2–59.1%), which was observed to reduce with each subsequent stage of embryo development, to the lowest degree of spatial overlap at the blastocyst stage (17.3%, 95% CI: 10.7–25.9%). Comparatively, explanations generated by ResNet-34 showed similar overlap between both XAI outputs from 2-cell embryos (28.7%, 95% CI: 22.3–35.0%) to 8 or 9-cell embryos (29.2%, 95% CI: 23.8–32.9%). Nonetheless, IoU values for ResNet-34 and VGG16 were comparable from the 4-cell stage to the blastocyst stage. Strikingly, despite both DL models perfectly classifying blastocyst stage embryos, the median IoU values for ResNet-34 and VGG16 are the lowest among all other embryo stages (*ResNet-34*: 20.2%, 95% CI: 18.5–27.3; *VGG16*: 17.3%, 95% CI: 10.7–25.9). Moreover, upon calculating the level of overlap among “poor” Grad-CAM explanations (Figure 5C), no spatial overlap was observed from VGG16 at the morula stage, suggesting the observed Grad-CAM explanations did not intersect with corresponding LIME super pixels from the same embryos.

## 4 Discussion

This study investigated differences in accuracy and inter-rater agreement between human operators and DL models during embryo stage classification. Embryologists, individually and collectively (89.9%), achieved greater accuracy in embryo stage classification compared to ResNet-34 (78.8%, *p* < 0.001) and VGG16 (74.3%, *p* < 0.001). Despite these observed differences in accuracy, all raters demonstrated excellent overall agreement with the reference standard, and with other operators. Contrastingly, further analyses conducted at the developmental stage level revealed lower agreement between DL models, thereby suggesting variations in underlying classification performance that may not be represented by accuracy assessments alone. Further divergence between models was detected upon assessing the biological relevance of Grad-CAM explanations. Although our findings indicate that Grad-CAM explanations generated by ResNet-34 were more frequently deemed biologically meaningful (89%) compared to their VGG16 Grad-CAM counterparts (59%, *p* < 0.001), trends of spatial overlap with LIME explanations suggest that the spatial focus of models may vary by embryo stage, and by the XAI method utilized. Taken together, these findings demonstrate that accuracy, agreement and interpretability are metrics that may not be consistently aligned. Instead, these performance indicators may represent distinct facets of model behavior that may be used to improve trustworthiness and further development of AI-driven assessment tools.

Although accuracy remains the most frequently reported metric within embryo classification models, clinical reliability is often established by measuring agreement between operators (56). Therefore, we evaluated inter-operator agreement using quadratic-weighted Cohen's kappa (*κ*) as an additional model performance indicator (57). Intriguingly, despite reduced classification accuracy by both DL models, their agreement to the reference standard remained high (*ResNet-34*: *κ* = 0.932, *p* < 0.001; *VGG16*: *κ* = 0.940, *p* < 0.001). Similarly, pairwise comparisons indicated excellent agreement between both DL models, and each individual embryologist. Based on this finding, we speculate the increased weighted *κ* may not be representative of the high proportions of adjacent-cell stage errors by DL models. Although the use of a quadratic-weighted Cohen's *κ* mirrors the ordinal nature of embryo development, this method of assessing agreement penalizes adjacent cell-stage errors *less* than misclassifications at more distant stages. This notion was reinforced upon investigating per-class agreement between operators, where DL models displayed moderate to fair agreement (*κ* range: 0.385–0.681) across embryo stages, compared to consistently high embryologist agreement (*κ* range: 0.778–0.952). Notably, the calculated *κ* value was unstable at the 2-cell and 3-cell stages, indicating this analysis may be underpowered and requires cautious interpretation. Nonetheless, this divergence revealed that while ResNet-34 and VGG16 achieve comparable classification accuracy, DL models may perform with different intrinsic reasoning that contributes towards unstable agreement (35). In fact, our findings align with another study reporting low inter-AI agreement (21), which remains, to the best of our knowledge, the only investigation reporting agreement metrics between AI-systems. Thus, unlike many studies that focus primarily on performance (12, 13, 58, 59), our results join recent efforts in highlighting the importance of understanding *how* models reach their decisions.

Recognizing the reduced agreement between ResNet-34 and VGG16 classifications, we further examined embryologists' assessment of Grad-CAM heatmaps to ascertain differences in model spatial attention. Overall, ResNet-34 Grad-CAM outputs were rated as biologically relevant more frequently (89%) compared to VGG16 generated heatmaps (59%, *p* < 0.001). Furthermore, consistently “good” assessments of ResNet-34 Grad-CAMs at the embryo stage-level implies that the model may prioritize features more congruent with embryologist decision-making than VGG16. However, this interpretive advantage was not always aligned with accuracy. At the 5-cell stage, 100% of ResNet-34 Grad-CAMs were rated as “good”, yet only 43% of classifications were correct, implying that well-rated explanations of a qualitative nature may not reliably reflect the features driving model predictions (51, 60, 61). Crucially, these findings raise the concern of interpretability without accuracy, and empirically validate several concerns raised by van Royen et al. (39) regarding the deployment of XAI in clinical settings. In fact, our results exemplify the risk of “illusory understanding” that arises when embryologists are presented with visually convincing *post-hoc* explanations that appear plausible, but may obscure underlying discrepancies in model behavior (62). In such cases, explanatory visualizations may actually impede clinical skepticism by overinterpretations of causality and an increased risk of confirmation bias (39). Conversely, VGG16 achieved high accuracy at the morula stage (94%) despite 70% of its Grad-CAM outputs being rated “poor,” thereby reflecting accuracy without interpretability. Despite a dearth of literature surrounding these discrepancies within embryo-related tasks, correct predictions that arise from spurious or non-biological features are known to undermine trust and implementation within a range of healthcare settings (60, 61, 63, 64). Importantly, the observed divergence in interpretability between ResNet-34 and VGG16 Grad-CAM outputs occurred despite similar overall accuracy, thus demonstrating that accuracy and interpretability may represent distinct dimensions of model behavior rather than interchangeable indicators of reliability. Taken together, our findings underscore the need for caution when integrating saliency-based XAI tools into embryo assessment workflows, where explanation quality metrics should be evaluated independently from model reliability, instead of a proxy for model performance. This highlights a meaningful gap between model performance and interpretive agreement that warrants further research in embryo-related tasks.

Further, we expanded our investigation to determine whether independent XAI methods, Grad-CAM and LIME, converged on salient features using IoU-based spatial overlap. Overall, we observed that ResNet-34 exhibited a greater degree of median overlap between Grad-CAM and LIME explanations (26.6%) compared to VGG16 (25.2%, *p* = 0.006). Upon stage-wise assessment, however, significant variations in median overlap were only observed at the morula (*ResNet-34*: 22.5% vs. *VGG16*: 0%, *p* < 0.001) and blastocyst stage (*ResNet-34*: 21.0% vs. *VGG16:* 17.0%, *p* = 0.006). Given that both models achieved high accuracy at these stages, the limited spatial overlap between explanations is unexpected and prompts reconsideration of whether saliency-based spatial attention serves as a reliable proxy for classification accuracy. Indeed, based on embryologists’ qualitative assessment of Grad-CAM outputs, the majority of VGG16 explanations at the morula stage were “poor”. Yet, the complete absence of overlap with LIME at this stage suggests that while Grad-CAM visualizations appeared uninformative to embryologists, LIME may have captured alternative, potentially relevant regions of interest. Even so, perfect classification accuracy at the blastocyst stage encapsulating mostly “good” Grad-CAM explanations, resulted in the lowest IoU for both models. Importantly, this highlights that XAI tools may capture different, potentially arbitrary, features of the same input (64, 65), rather than converging on causal factors driving classification decisions. Furthermore, this raises concerns about whether XAI methodologies offer genuine mechanistic insight that is grounded in model reasoning, or simply provide a veneer of interpretability that satisfies human intuitions (63). Further, we speculate that that within the range of developmental stages explored here, the morula and blastocyst present with the most distinct embryo morphologies, at relatively low numbers. This raises concerns regarding overfitting, which may have also contributed to their increased accuracy (66). It is also important to acknowledge that LIME is inherently stochastic, such that super pixel boundaries may vary across runs, thereby limiting the stability of IoU-based comparisons (67, 68). Collectively, these findings illustrate that agreement between *post-hoc* XAI methods is not necessarily informed by accuracy but may prove useful in the enhancement of human-perceived interpretability.

Collectively, this work provides a systematic evaluation of accuracy, agreement and interpretability within embryo-stage classification, demonstrating that each component represents an independent dimension of model performance that may diverge within practical tasks. Notably, we highlight that accuracy may arise without biologically grounded interpretive focus, but also that observed interpretive focus lacks application-based and functionality-grounded evaluation (69). Granting clinical reliability remains largely based on inter-operator agreement, our findings highlight how agreement metrics can quantitatively and qualitatively vary between equally performing models (35). To our knowledge, this is the first investigation to evaluate embryo-level XAI outputs through direct embryologist review, thereby establishing domain expert focus within interpretability assessments, rather than model performance metrics alone. Notwithstanding, this study harbours several limitations. First, only single-frame images were extracted from retrospectively annotated TLT recordings for the purpose of classification, which may not fully capture the complexity and variability of dynamic embryo development. Additionally, the inclusion of embryos with the most complete morphokinetic annotations upon data collection, in comparison to arrested embryos for example, may have introduced selection bias. Moreover, subjective evaluation of Grad-CAM explanations by embryologists may have introduced confirmation bias towards the biological relevance of generated heatmaps. Similarly, although morphological classification is strongly associated with reproductive potential, it is not equivalent to the primary end-goal of live birth, which limits its clinical generalizability. As such, further research should prioritize the use of large, multicenter datasets to quantitatively and qualitatively interpret model behaviors that are tailored towards the intended domain. For example, as studies strive to create objective measures of explainability, the quantitative use of saliency map-based segmentation shows promise within user interpretability (35, 60, 70). However, our findings caution the calibration of trust within AI predictions based on the perceived quality of model explanations alone, as the observed dissociation between XAI interpretability and accuracy may, paradoxically, lead to further compromised decision-making than “black box” models. Overall, establishing robust standards and alignment between accuracy, agreement and interpretability will be essential for the responsible implementation of AI-tools within IVF laboratories.

## Data Availability

The raw data supporting the conclusions of this article will be made available by the authors, without undue reservation.
