# Supplementary material for: Investigating discrepancies in accuracy, agreement and interpretability for single-frame embryo classification tasks conducted by embryologists and deep learning models
Source: Front Reprod Health. 2026 Mar 3;8:1778326. doi: 10.3389/frph.2026.1778326 (PMC13040276; doi:10.3389/frph.2026.1778326)
Supplement: Supplementary file 1 [file Table1.docx]

Supplementary Material

**Supplementary Table 1**: Performance of DL models based on quantitative assessment of six performance metrics, as described in Sharma et al (2022). Recall (REC), precision (PREC), specificity (SPEC), accuracy (ACC), Matthews correlation coefficient (MCC) and F1-score are reported. MCC reports the difference between actual and predicted labels, providing a value between -1 and 1, where a value above zero and closer to 1 indicates a greater number of correct predictions. The F1-score is a weighted average of PREC and sensitivity (REC) which reports the models’ performance of false positives and false negatives between 0 to 1.

|  | | **Independent Test Dataset** | | | | | | | | | |
| --- | --- | --- | --- | --- | --- | --- | --- | --- | --- | --- | --- |
| **Models** | | REC | PREC | | SPEC | | ACC | | MCC | | F1 |
|  | VGG16 | 0.673 | 0.684 | 0.953 | | 0.673 | | 0.630 | | 0.665 | |
|  | ResNet-34 | 0.702 | 0.702 | 0.957 | | 0.702 | | 0.662 | | 0.693 | |

**Supplementary Material 2: Qualitative evaluation of Grad-CAM outputs.** The following information was provided to embryologists assessing generated Grad-CAM explanations. This material was constructed following several focus groups with clinical embryologists.

**ResNet34, VGG16: Grad-CAM Qualitative Evaluation**

To qualitatively assess the best performing models, the features used to classify each image are visualized using Gradient-weighted Class Activation Mapping (Grad-CAM).

Heatmaps are generated to visualize the embryo features that were discriminative for classification in the independent test (IT) dataset alone. The resultant color grading ranges from areas of **high relevance** (**red**) to **low relevance** (**blue**), for each model.

Your task is to assess each image, according to the following criteria:

| **Evaluation** | **Description** |
| --- | --- |
| **Good (A1-A2)** | *The model has identified areas of high relevance (red zones) which are localized within biologically relevant structures or areas* |
| **Poor (C1-C2)** | *The model has identified areas of high relevance (red zones) which do not correspond to the correct, or any, biologically relevant structures or areas* |
| **Intermediate (B1-B2)** | *The model has identified areas of high relevance (red zones) that are sometimes localized to biologically relevant structures or areas but also identify incorrect or biologically irrelevant structures or areas.* |

There are 490 Grad-CAM images in total, that represent the 2-cell, 3-cell, 4-cell, 5-cell, 8-cell, 9+ cell, morula and blastocyst stages. All images have been randomized amongst cell stages, as well as the model in question (ResNet34 or VGG16). Please only select one evaluation criteria (good, poor or intermediate) for each presented image.

**
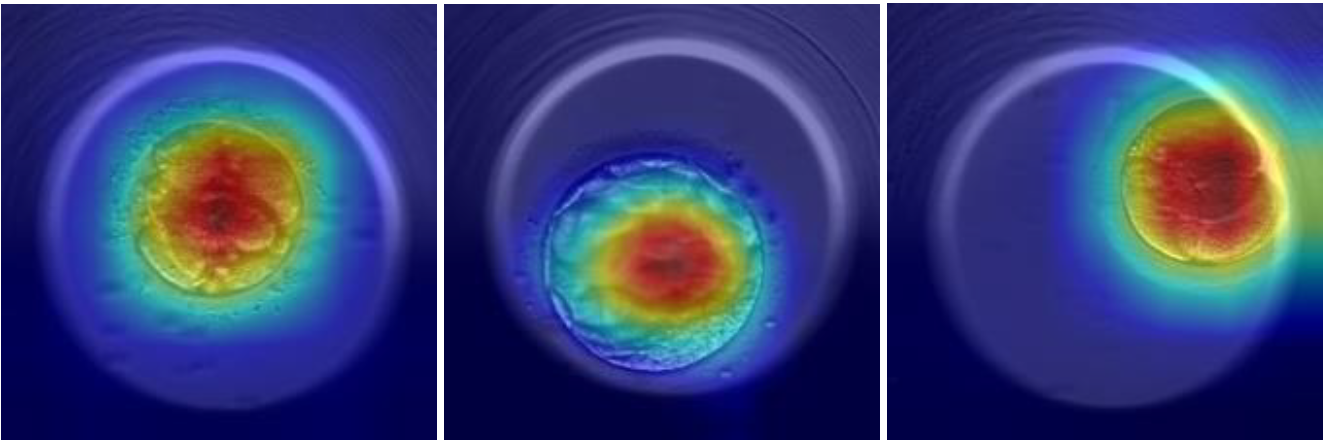
A1:** Centrally focused on/within the entire embryo. Most red-coloured high relevance zone(s) fall within the centre of the embryo, while the cyan-coloured low relevance zone(s) approximately align with the zona pellucida or peripheral boundaries of the embryo.

**
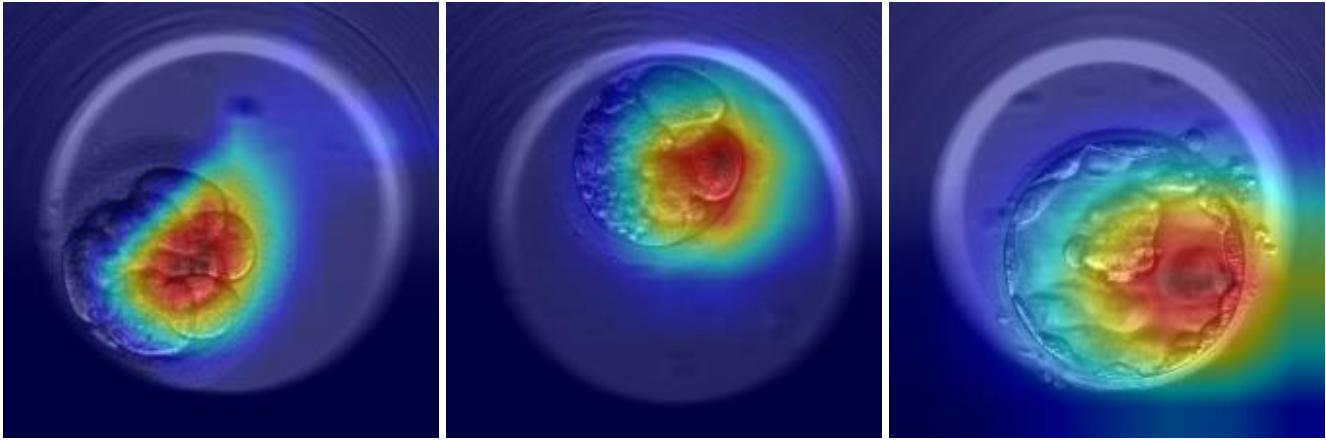
A2:** Focused peripherally or off-centre on/within the embryo. Most red-coloured high relevance zone(s) are found off-centre, where the focus seems asymmetric relative to the entire embryo. It may appear that the focus is on one or several cells only, as opposed to a central location within the embryo. Meanwhile, cyan-coloured low relevance zone(s) do not coincide with the approximate location of the zona pellucida and may appear outside the boundaries of the embryo. In some instances, a “colour run” may be observed. This is depicted by a streak of yellow, green and/or cyan-coloured zones that seem to “run” off the edges of the embryo, and the image itself (e.g. Panel 3, left to right).

**
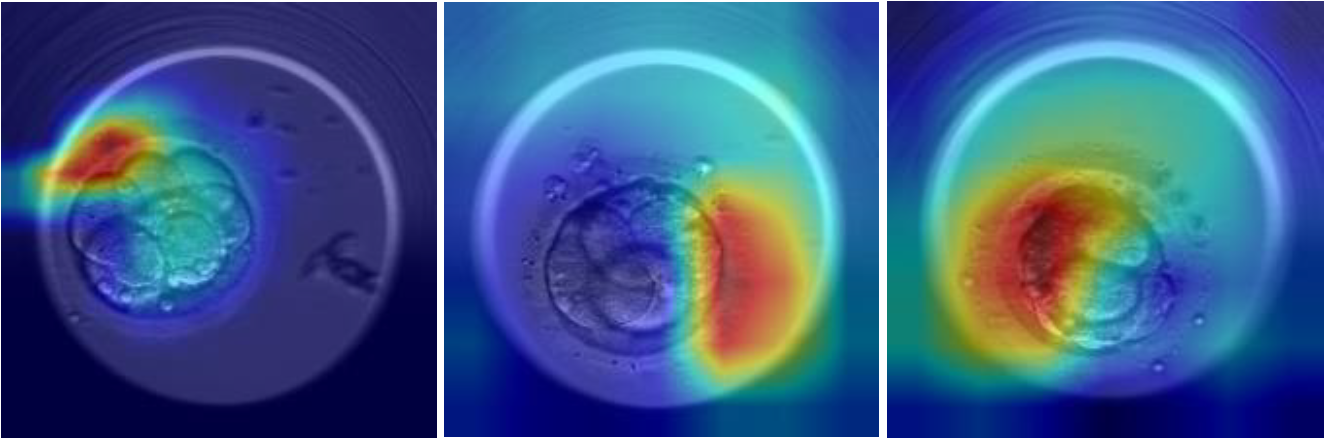
B1**: Focused on boundaries/edges of the embryo or zona pellucida only, with no obvious red-coloured areas on individual cells within the embryo. Cyan-coloured zones may appear centrally within the embryo or diffusely surrounding the entire image.

**
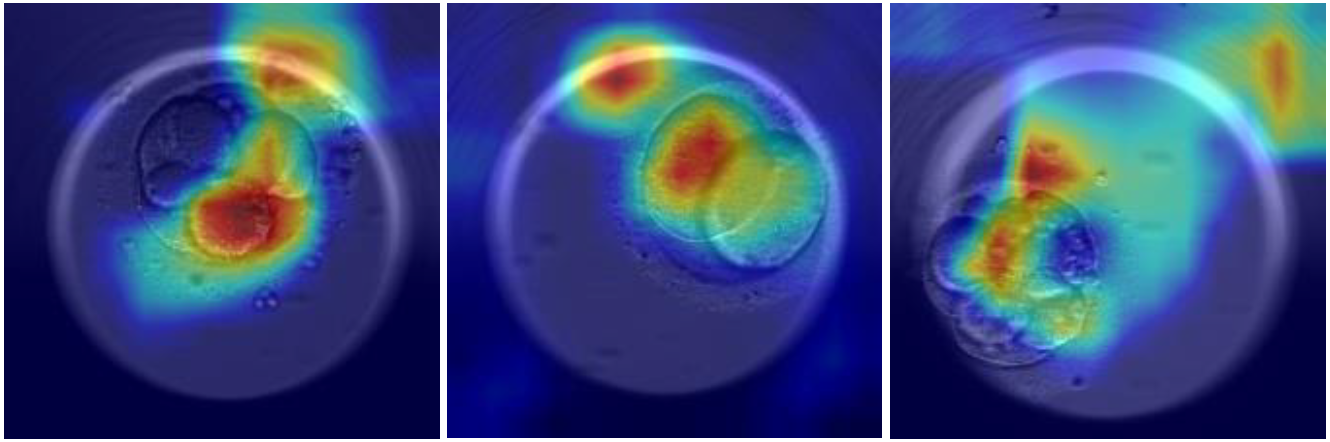
B2**: Although some cells within the embryo proper are identified as highly relevant red zones, other areas outside of the embryo, such as granulosa cells, the boundary of the well, or the well walls are also identified as highly relevant red zones. Cyan-coloured zones may appear centrally within the embryo or diffusely surrounding the entire image.


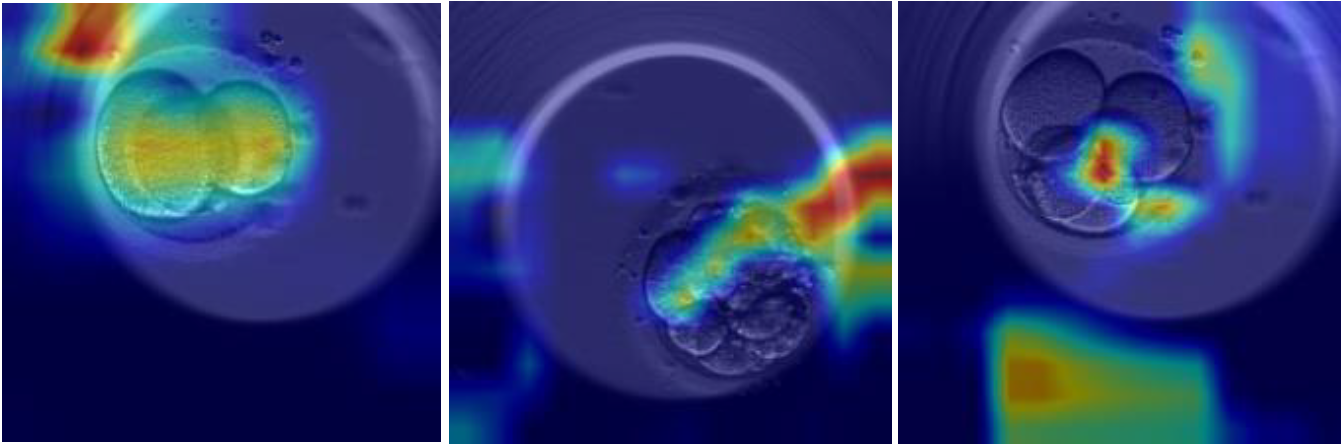
**C1**: Red zones are identified very faintly within biologically relevant structures within the embryo and are primarily located on biologically irrelevant structures outside the embryo such as the boundary of the well, or the well wall.

**C2**: Red zones are solely identified outside the boundaries of the embryo and are only found on biologically irrelevant structures outside the embryo such as the boundary of the well, or the well wall.


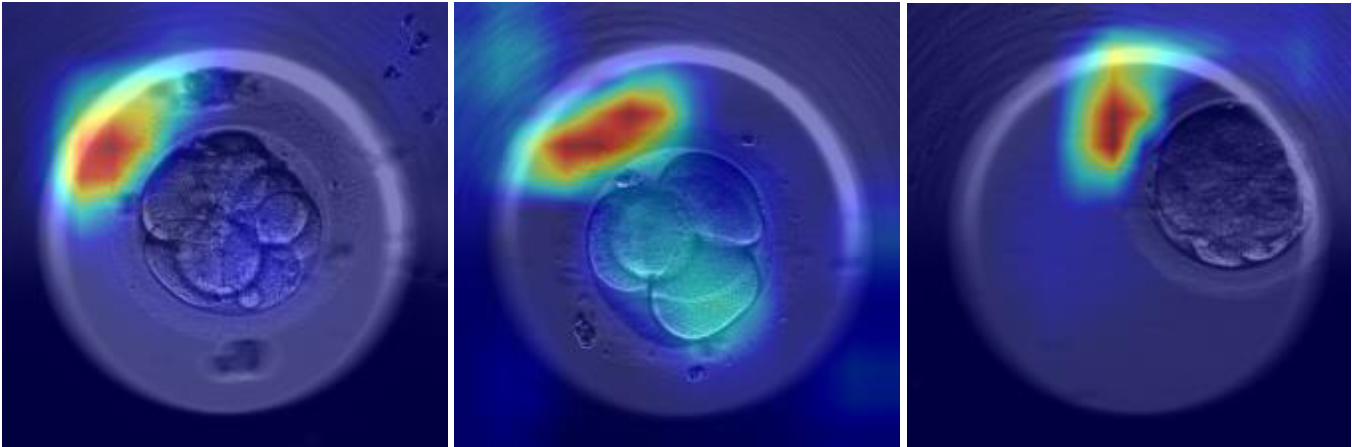


**Supplementary Table 3: Stage-wise agreement among human and model operators.** Fleiss’ multirater kappa (κ) was calculated to assess the agreement among embryologists, as more than 2 operators were present. To assess agreement between the deep learning (DL) models, Cohen’s kappa (κ) was used as only two operators were being compared. 95% confidence intervals (CI) are reported along with the corresponding p-value for each stage of embryo development. P-values less than 0.05 are considered significant.

| Embryologists | Stages | κ | 95%CI | p-value |
| --- | --- | --- | --- | --- |
|  | 2-cell | 0.952 | (1.024-0.879) | <0.001 |
|  | 3-cell | 0.778 | (0.851-0.706) | <0.001 |
|  | 4-cell | 0.780 | (0.852-0.708) | <0.001 |
|  | 5-cell | 0.798 | (0.870-0.726) | <0.001 |
|  | 8 or 9-cell | 0.904 | (0.976-0.831) | <0.001 |
|  | Morula | 0.929 | (1.000-0.877) | <0.001 |
| DL models | 2-cell | -0.054 | (0.034 - -0.143) | 0.696 |
|  | 3-cell | -0.005 | (0.485 - -0.496) | 0.975 |
|  | 4-cell | 0.427 | (0.640 – 0.214) | 0.015 |
|  | 5-cell | 0.681 | (0.860 – 0.503) | <0.001 |
|  | 8 or 9-cell | 0.385 | (0.765 – 0.004) | 0.003 |
|  | Morula | 0.576 | (1.22 - -0.070) | <0.001 |

**Supplementary Table 4: Assessment of Grad-CAM explanations by embryologists.** A total of 490 Grad-CAM outputs (n=245 per model) were evaluated for biological relevance as “good”, “intermediate” or “poor”. Actual counts are presented for each category, at each stage of development, with proportions listed in parentheses. Number of images in each developmental stage category are listed. Corresponding *p*-values are calculated using chi-squared analyses reflect statistically significant differences in proportions between ResNet-34 and VGG16 generated explanations. Differences were considered significant if *p*<0.05 and are presented in bold typeface.

|  | **Qualitative assessment by embryologists** | | | | | | | | |
| --- | --- | --- | --- | --- | --- | --- | --- | --- | --- |
| **Stage** | **Good** | | | **Intermediate** | | | **Poor** | | |
|  | *ResNet* | *VGG* | *P-value* | *ResNet* | *VGG* | *P-value* | *ResNet* | *VGG* | *P-value* |
| 2-cell (n=31) | 31  (100%) | 12 (38.7%) | **<0.001** | 0  (0%) | 4 (12.9%) | 0.113 | 0  (0%) | 15 (48.4%) | **<0.001** |
| 3-cell (n=31) | 29 (93.5%) | 18 (58.1%) | **0.002** | 1  (3.2%) | 4 (12.9%) | 0.354 | 1 (3.2%) | 9 (29%) | **0.013** |
| 4-cell (n=32) | 31 (96.9%) | 20 (62.5%) | **0.001** | 0  (0%) | 4 (12.5%) | 0.113 | 1 (3.1%) | 8 (25%) | **0.027** |
| 5-cell (n=29) | 29  (100%) | 20 (69%) | **0.002** | 0  (0%) | 5 (17.2%) | 0.052 | 0  (0%) | 4 (13.8%) | 0.112 |
| 8-9 cells (n=61) | 56 (91.8%) | 41 (67.2%) | **0.001** | 5  (8.2%) | 12 (19.7%) | 0.114 | 0  (0%) | 8 (13.1%) | **0.006** |
| Morula (n=30) | 16 (53.3%) | 6  (20%) | **0.015** | 13 (43.3%) | 3  (10%) | **0.007** | 1 (3.3%) | 21  (70%) | **<0.001** |
| Blastocyst (n=31) | 25 (80.6%) | 28 (90.3%) | 0.473 | 6 (19.4%) | 3  (9.7%) | 0.473 | 0 (0%) | 0 (0%) | >0.999 |
